# Supplementary material for: Laser-induced thermal source for cold atoms
Source: Sci Rep. 2022 Jan 18;12:868. doi: 10.1038/s41598-021-04697-4 (PMC8766515; doi:10.1038/s41598-021-04697-4)
Supplement: Supplementary file 1 — Supplementary Information. [file 41598_2021_4697_MOESM1_ESM.pdf]

Supplementary Information: Laser-induced thermal source for cold atoms

Chung Chuan Hsu[1], Rémy Larue[2,3], Chang Chi Kwong[1,3,*], and David Wilkowski[1,3,4]

[1]School of Physical and Mathematical Science, Nanyang Technological University, 637371 Singapore, Singapore
[2]Laboratoire de Physique Subatomique et de Cosmologie, Université Grenoble-Alpes, CNRS/IN2P3, Grenoble INP, 38000 Grenoble, France
[3]MajuLab, International Research Laboratory, IRL 3654, CNRS, Université Côte d'Azur, Sorbonne Université, National University of Singapore, Nanyang Technological University, Singapore.
[4]Centre for Quantum Technologies, National University of Singapore, 117543 Singapore, Singapore
[*]changchikwong@ntu.edu.sg

Here, we describe how the number of atoms in the MOT is approximated from the fluorescence signal captured by a system with a detection solid angle of Ω. Our work is done only on the ^{88}Sr isotope. The cooling transition is a $J = 0 \to J = 1$ transition, where scattering cross section is the same for all laser polarizations. We start by considering a single atom and calculate P_a, which is the fluorescence power emitted by a single strontium atom into the solid angle of Ω:

$$P_a = E_p \Gamma' \frac{\Omega}{4\pi} \tag{1}$$

Here, $E_p = hc/\lambda$ is the energy of a photon at $\lambda = 461$ nm, and Γ' denotes the rate of number of photons scattered per atom. We denote the speed of light by c and Planck's constant by h. We consider P_a to be averaged over many fluorescence events of all the atoms in the MOT and assume that its angular distribution is isotropic. Since the natural linewidth is $\Gamma = 32 \times 2\pi$ MHz,

$$\Gamma' = \frac{1}{2}\frac{s}{1+s}\Gamma = \frac{1}{2}\frac{s_0}{4\delta^2/\Gamma^2 + 1 + s_0}\Gamma \tag{2}$$

where $s = s_0/(4\delta^2/\Gamma^2 + 1)$, δ is the detuning of the MOT beams, $s_0 = I_L/I_S$ is the saturation parameter and I_S is the saturation intensity of the cooling transition, which is 43 mW/cm^2. For our MOT beams with a total power of P and a waist of w, the total intensity is $I = 2P/\pi w^2$. For small detection angles, we perform the approximation $\Omega/4\pi \approx \frac{1}{2}(1 - \cos\frac{D}{2d})$, where the camera lens diameter is D and the imaging distance is d. This leads to the following expression for P_a

$$P_a = E_p \Gamma' \frac{\Omega}{4\pi} = \left(\frac{hc}{\lambda}\right)\left(\frac{1}{2}\frac{s_0}{4\delta^2/\Gamma^2 + 1 + s_0}\Gamma\right)\left[\frac{1}{2}\left(1 - \cos\frac{D}{2d}\right)\right] \tag{3}$$

Finally, we divide the total MOT power detected by the camera P_T by P_a such that number of atoms is therefore $N = P_T/P_a$. P_T was pre-determined via calibrating the counts to a known beam power. Using this formula, we are able to estimate the number of atoms for any given fluorescence data captured by the CMOS camera in our experiments.
